# Biomarkers of animal health: integrating nutritional ecology, endocrine ecophysiology, ecoimmunology, and geospatial ecology

**DOI:** 10.1002/ece3.1360

**Published:** 2015-01-07

**Authors:** Robin W Warne, Glenn A Proudfoot, Erica J Crespi

**Affiliations:** Department of Biology, Vassar College124 Raymond Ave, Poughkeepsie, New York, 12604

**Keywords:** Bioindicators, birds, glucocorticoids, isoscapes, migration, stable isotopes, stress physiology

## Abstract

Diverse biomarkers including stable isotope, hormonal, and ecoimmunological assays are powerful tools to assess animal condition. However, an integrative approach is necessary to provide the context essential to understanding how biomarkers reveal animal health in varied ecological conditions. A barrier to such integration is a general lack of awareness of how shared extraction methods from across fields can provide material from the same animal tissues for diverse biomarker assays. In addition, the use of shared methods for extracting differing tissue fractions can also provide biomarkers for how animal health varies across time. Specifically, no study has explicitly illustrated the depth and breadth of spacial and temporal information that can be derived from coupled biomarker assessments on two easily collected tissues: blood and feathers or hair. This study used integrated measures of glucocorticoids, stable isotopes, and parasite loads in the feathers and blood of fall-migrating Northern saw-whet owls (*Aegolius acadicus*) to illustrate the wealth of knowledge about animal health and ecology across both time and space. In feathers, we assayed deuterium (*δ*D) isotope and corticosterone (CORT) profiles, while in blood we measured CORT and blood parasite levels. We found that while earlier migrating owls had elevated CORT levels relative to later migrating birds, there was also a disassociation between plasma and feather CORT, and blood parasite loads. These results demonstrate how these tissues integrate time periods from weeks to seasons and reflect energetic demands during differing life stages. Taken together, these findings illustrate the potential for integrating diverse biomarkers to assess interactions between environmental factors and animal health across varied time periods without the necessity of continually recapturing and tracking individuals. Combining biomarkers from diverse research fields into an integrated framework hold great promise for advancing our understanding of environmental effects on animal health.

## Introduction

Examining landscape scale and interannual effects of environmental variation on animal health at both individual and population levels is generally a challenging prospect, limited by our capacity to track and monitor animal physiological states and responses across space and time. This is particularly true for highly mobile and migrating animals. However, monitoring animals and their responses to ecologically relevant conditions are central to understanding how they cope with and adapt to changing environments. To meet this challenge numerous fields of biological research have endeavored to identify and develop biomarkers for organismal function that include endocrine, immune, nutritional, metabolic, and behavioral processes. Because these systems are interdependent components of physiological regulatory networks, there has also been a growing recognition that an integrative approach is necessary to contextualize and interpret the relevance of any given biomarker profile (Martin et al. 2011; Cohen et al. 2012; Milot et al. 2014).

Two examples of integrated physiological biomarkers are glucocorticoid (GC) concentrations and stable isotopes. Considering that the endocrine system helps to mediate physiological and behavioral responses to external stimuli, the field of ecological endocrinology has developed a rich knowledge base of how hormones such as GCs can, to some extent, be used as indicators of animal states and responses to changing environmental conditions (McGlothlin and Ketterson 2008; Wingfield et al. 2008). But, more recently, it has become clear that to understand the importance of GC levels for the condition and health of animals, these biomarker profiles must be linked in a more holistic way to interrelated organismal functions and ecological context (McGlothlin and Ketterson 2008; Bonier et al. 2009; Romero et al. 2009; Milot et al. 2014). Similarly, the field of stable isotope physiological ecology has developed the use of naturally occurring isotopes, such as *δ*^13^Carbon, *δ*^15^Nitrogen, and *δ*Deuterium as indicators of animal nutritional and metabolic processes (Hobson and Clark 1993; Wolf et al. 2009; Warne et al. 2010a, 2012), as well as their responses to ecological and landscape scale dynamics (Hobson et al. 1994; Hobson 1999; Bowen 2010a; Warne et al. 2010b). However, to use stable isotopes as biomarkers, it is also necessary to link their profiles to whole-organism function and ecological conditions (Gannes et al. 1997; Wolf et al. 2009). These findings from separate but interrelated fields demonstrate that there is both a great need as well opportunity to develop integrated methods and biomarkers to better detail animal condition and health.

**Figure 1:**
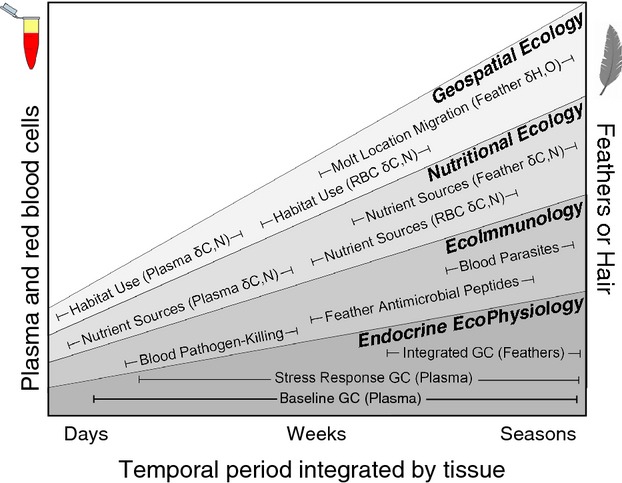
A diversity of biomarkers derived from numerous integrative fields can be assayed on blood and feathers (or other keratinaceous tissues) collected from the same individual. By applying these assays to the same tissues, differing time periods (days, weeks to seasons) can be examined, and a more comprehensive assessment can be developed for the state and health of animals, as well as how a host of ecological factors affects them. Note that the order in which the fields are presented is random, but the positive trend of each shape suggests the increasing depth of information that can be gained through multiple assays applied to the same tissues.

A barrier to integrating the diverse biomarkers available is a general lack of awareness and knowledge of the availability, collection, and use of markers common to a given field. In particular, we find that it is not common knowledge, nor has there been a deliberate demonstration of the depth and breadth of information that can be derived from just two easily collected tissues: blood and feathers or hair (Fig. 1). From these two tissues, information can be derived on hormone profiles, immune function, nutrient allocation, as well as geospatial and ecological interactions. In addition, because these tissues as well as their differing constituents are grown or turnover at varying rates (Bortolotti et al. 2009; Wolf et al. 2009; Warne et al. 2010a), a breadth of temporal information can be gleaned from an integrative analysis. For example, a single sample of blood can be used to measure dietary sources at both week and month long intervals through *δ*^13^Carbon analysis of plasma and red blood cell fractions (Warne et al. 2010a). From this same sample, aliquots of whole blood can also be used for immunity measures such as microparasite loads and blood-pathogen killing capacity; both of which are broad spectrum measures of immune function at monthly to seasonal timescales (Liebl and Martin 2009; Matson et al. 2012). In addition, baseline and stress-induced GC levels can also be measured in these same or serial blood samples, which can provide insight into the relative energetic “workload” borne by an animal (Bonier et al. 2011; Crespi et al. 2013; Jenkins et al. 2013; Deviche et al. 2014). While GCs are often thought of as stress hormones, a consensus is growing around a more nuanced concept of these as indicators of energy allocation and mobilization that reflect the state of an animal across life stages, and predictable long-term seasonal cycles, as well during short-term unexpected perturbations (Wingfield et al. 2008; Bonier et al. 2009; Boonstra 2013; Crespi et al. 2013). Feathers or hair can also be collected from the same individual to provide complementary data (Fairhurst et al. 2013a). While feathers are commonly analyzed for *δ* Deuterium or *δ*^18^Oxygen isotopes for geospatial analysis of breeding and wintering grounds in migrating birds (Hobson et al. 2004; Bowen et al. 2005), it is less commonly known that the oils/lipids that must be removed from these feathers prior to isotope analysis also contain GC hormones that are integrated into the feather at the time of growth (Bortolotti et al. 2008, 2009). Thus, a suite of assays can be run on differing fractions of two tissues from the same animal to provide a broader context for biomarkers and potentially a deeper understanding of an animal's condition and health over both short and longer timescales (Fig. 1).

Here, we provide an illustration and overview of this integrative approach through assaying a suite of biomarkers in fall-migrating Northern saw-whet owls (*Aegolius acadicus*). We assayed GC hormone profiles in blood and feathers, blood parasite loads, and *δ* Deuterium profiles to deduce the breeding origins of eastern populations of this owl. We hypothesized that because Northern saw-whet owls take ∽21 days to grow tail feathers (G. Proudfoot, unpublished data), that GC hormone levels in these feathers would provide an integrated measure of energetic condition in these owls during the end of breeding and the period of costly preparation for migration. In contrast, blood GC levels should reflect the energetic-workload conditions of individual owls during migration. We also examined interactions among GC levels and body condition and blood parasite loads, because these can influence energy expenditure. In addition, saw-whet owls are an interesting species to explore these concepts, because across their range they exhibit boom and bust years with great interannual variation in the total number of birds migrating, their sex ratio, and age distribution, which may reflect environmental variation in resource density (Whalen and Watts 2002; Beckett and Proudfoot 2011). While we do not intend to provide an exhaustive review of the diverse assays available, our aim was to provide an overview and illustration of how the integration of such biomarkers can shed light on whole-organism function within the context of environmental conditions.

## Methods

### Capture and sampling

Northern saw-whet owls (*n* = 209) were captured by audio-lure mist-netting (Erdman and Brinker 1997) at Mohonk Preserve in Ulster County, NY from 1 October to 2 December, 2011. Each owl was fitted with a U.S. Geological Survey aluminum leg band, weighed, measured for wing chord and tail length, and aged by molt patterns of primary and secondary feathers (Evans and Rosenfield 1987). Approximately 200 *μ*L of whole blood was collected from the dorsal pedal artery of each owl using 0.5 cc insulin syringes (29 gauge) and two heparinized capillary tubes. In one tube, plasma and red blood cell fractions were separated by centrifugation, and the plasma was transferred to eppendorf tubes (100 *μ*L) for storage at −20°C and later hormone analysis. Approximately 5 *μ*L of whole blood was used to create a thin blood smear on a microscopy slide. Each slide was labeled with the corresponding owl's band number. The blood smears were air dried and immediately fixed with 100% methanol. The remaining blood was stored in 2 mL cryotubes (ABgene, Thermo Fisher Scientific, Inc., Waltham, MA) with 1 mL of preservation buffer and was used later to assign sex using molecular analysis (Longmire et al. 1988). In addition, one tail feather, the outermost rectrice (R6) was plucked from each owl and stored in paper envelopes for later hormone analysis.

### Glucocorticoid assays of feathers and blood

To analyze corticosterone levels in owl feathers, the primary glucocorticoid in birds, one tail feather per individual (*n* = 131) was weighed and measured, after the calamus was discarded. The remaining feather was then cut into 5 mm pieces, and lipophilic substances (including GCs) were extracted following the protocol of Bortolotti et al. (2008). These clippings were vortexed in borosilicate tubes with 8 mL of methanol and then sonicated for 30 min in a sonicating water bath, followed by an overnight incubation in a 50°C water bath. The methanol was then decanted into clean tubes, and the cleaned feather remnants were air dried and used for stable isotope analysis (see below). The decanted methanol was evaporated in a ReactiVap at 50°C under nitrogen in a fume hood, and the resulting extract residue was then resuspended in 300 *μ*L of EIA buffer (from Cayman kits). The tubes were vortexed and warmed to 37°C for 10 min to ensure resuspension and then frozen at −20°C until EIA analysis.

To extract corticosterone from owl blood plasma, the lipophilic portions of the plasma were extracted using C18 solid-phase extraction (SPE) chromatography columns (3 mL columns; Thermo-Fisher Scientific). To achieve a dilution factor of 200 that had been validated to be optimal for detection in EIA assays (see below), 5 *μ*L of plasma was diluted in 795 *μ*L ultrapure water and then extracted in the SPE columns under vacuum (Wong et al. 2008; Gabor et al. 2013). Briefly, the columns were primed with HPLC grade methanol and Millipore filtered water, then the 800 mL of diluted plasma was filtered through the columns under vacuum. The extracted fraction was then eluted from these columns with 4 mL of methanol. The eluted methanol was then evaporated in a ReactiVap at 50°C under nitrogen in a fume hood, and the resulting extract residue was then resuspended in 300 *μ*L of EIA buffer (from Cayman kits).

Corticosterone (CORT) hormone levels in both feathers and blood were measured separately using enzyme-immunoassay (EIA) kits (Cayman Chemical Company, Ann Arbor, MI). Both tissues were validated in these kits using pooled samples for serial dilutions and quantitative recovery (Earley and Hsu 2008; Gabor et al. 2013). For the feather CORT, the serial dilution curve was parallel to the standard curve (ANCOVA slope comparison; *F*_1,9_ = 0.11, *P* = 0.75) and a dilution of 1:8 provided an optimal detection range. To examine recovery, the pooled feather samples were cold spiked in equal volume with each of the eight standards provided in the EIA kit (Earley and Hsu 2008; Gabor et al. 2013). Based upon comparison of these spiked standards to an unmanipulated pooled sample, the minimum recovery was 61%. Four plates were used for the feathers, with an interassay coefficient of variation (CV) based upon lab standards of 9%, and mean intra-assay CV of 5%. For plasma, the serial dilution curve was parallel to the standard curve (ANCOVA slope comparison; *F*_1,6_ = 0.28, *P* = 0.62) and a dilution of 1:200 provided an optimal detection range. The minimum recovery for plasma was 66%. Three plates were used for plasma, with an interassay (CV) of 8%, and mean intra-assay CV of 5%.

### Stable-hydrogen isotope analysis of feathers

For deuterium stable isotope analysis of the feathers, we used the feather remnants that had been cleaned of oil residues for the CORT extraction (see above). Clippings of these cleaned remnants were loaded at ∽0.25 mg into silver capsules (3 × 5 mm, Costech Analytical Technologies Inc., Valencia, CA) and analyzed at the Southern Illinois University Mass Spectrometry Facility. The samples along with the keratin standards CBS (Caribbou Hoof Standard), KHS (Kudu Horn Standard), and industrial keratin powder, as a quality control standard, were prepared for isotope analysis using a variation of the ambient equilibration with atmospheric method of Qi and Coplen (2011) and the comparative equilibration method with atmospheric moisture of Wassenaar and Hobson (2003). Briefly, in order to account for atmospherically exchangeable hydrogen present in the keratin samples, together with the more abundant nonexchangeable hydrogen, the feather samples and standards were left to equilibrate with atmospheric moisture for 2 weeks. These were then placed in a desiccator for 4 days and then left in a vacuum oven at room temperature (25°C) for 1 week.

These equilibrated samples and standards were then rapidly loaded into a Costech Zero Blank autosampler. They were pyrolized at 1420°C using a Finnigan Thermal Combustion Elemental Analyzer (TCEA) connected to a Thermo Scientific Finnigan Delta V Plus Isotope Ratio Mass Spectrometer (IRMS) with a GC column temperature of 78°C. Based upon the standards, the average analytical precision for *δ*D was ±2.7‰ KHS.

### Blood parasite assessment

Blood smears were stained as described by Bennett (1970) and were examined under Nikon Eclipse E600 (Nikon Inc., Melville, NY) optical microscopes. Each smear was examined for 100 fields at 200× magnification to assess prevalence of macro parasites (*Leucocytozoon* spp., microfilaria, and *Trypanosoma* spp.), and again (100 fields) with oil immersion at 1000× magnification to assess prevalence of micro parasites (*Haemoproteus* spp. and *Plasmodium* spp.) (Merino et al. 2000). Parasites were identified according to Valkiūnas et al. (2005) and photographed using SPOT Advanced 5.1 software (SPOTTM Imaging Solutions, Sterling Heights, MI).

### Statistical analysis

To estimate the locality at which the captured saw-whet owls had grown their feathers, we used the IsoMAP online workspace to produce isoscapes based upon our *δ*D results (Bowen et al. 2013; - Job #18356). IsoMAP uses known stable isotope ratio variation in the natural environment (Welker 2000; IAEA/WMO 2011) combined with a suite of web-based GIS and software tools to model and statistically predict geographically related isotope data (Bowen 2010b). General linear models were used to analyze the CORT results. For the response variable of feather CORT, the fixed factors included in the model were capture date, age, body condition, total parasite load, and plasma CORT. This full model was then reduced to capture date and age along with their interaction. For the response variable of plasma CORT, the fixed factors included in the model were capture date, age, body condition, and total parasite load.

## Results

### Stable-hydrogen isotope: breeding locations

**Figure 2:**
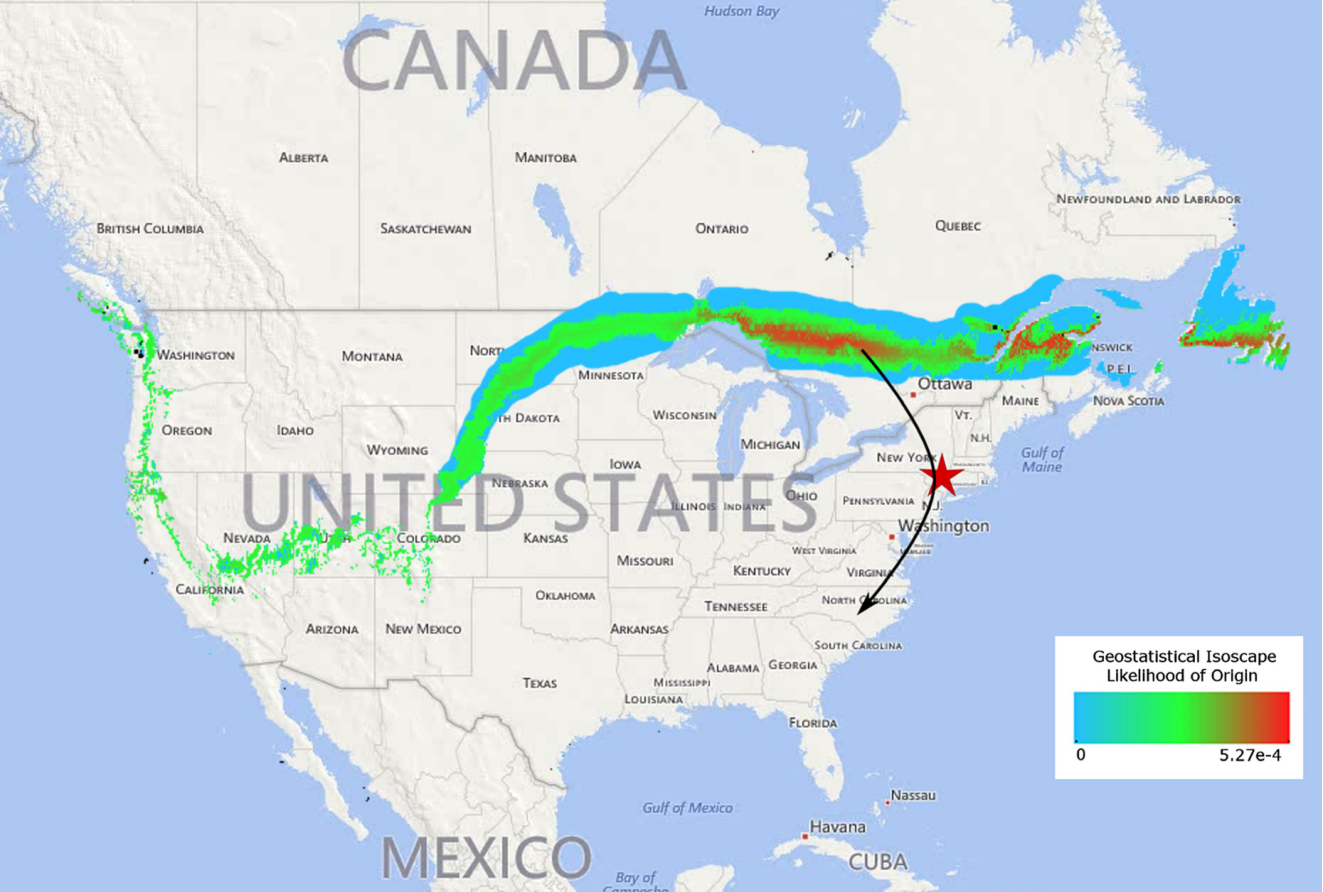
Geostatistical isoscape estimates for the origin of Northern saw-whet owls that were netted and banded in the Hudson Valley of New York. Estimates are based upon *δ*^2^Hydrogen analysis of feathers of hatch-year owls. IsoMAP Project: Isoscapes Modeling, Analysis, and Prediction (version 1.0). Available at: http://isomap.org.

We measured the *δ*D values of tail feathers for 125 Northern saw-whet owls migrating through the Hudson Valley of New York (Fig. 2). The feather *δ*D values differed among the age classes (*F*_1,119_ = 78.7, *P* < 0.001) and ranged from −40 to −93‰ VSMOW in hatch-year owls (*n* = 58) and −7 to −75‰ in adult owls (*n* = 67). Based on *δ*D values of hatch-year birds only, the breeding-fall molt locations for these owls were estimated to be along the southeastern border of Canada in the states of Ontario and Quebec (Fig. 2; Geostatistical Likelihood *P* < 0.005). Adults were not used in this geostatistical model because of irregular *δ*D values, which are values outside the range of known environmental *δ*D values for sources that include precipitation and ground water. This result is common in feather stable isotope analyses of raptors, and thus, juveniles are most often used in isotope analysis for migration (Smith et al. 2003; Smith and Dufty 2005; Gow et al. 2012), including Northern saw-whet owls (Ruyck et al. 2013).

### Glucocorticoid profiles and blood parasites

**Figure 3:**
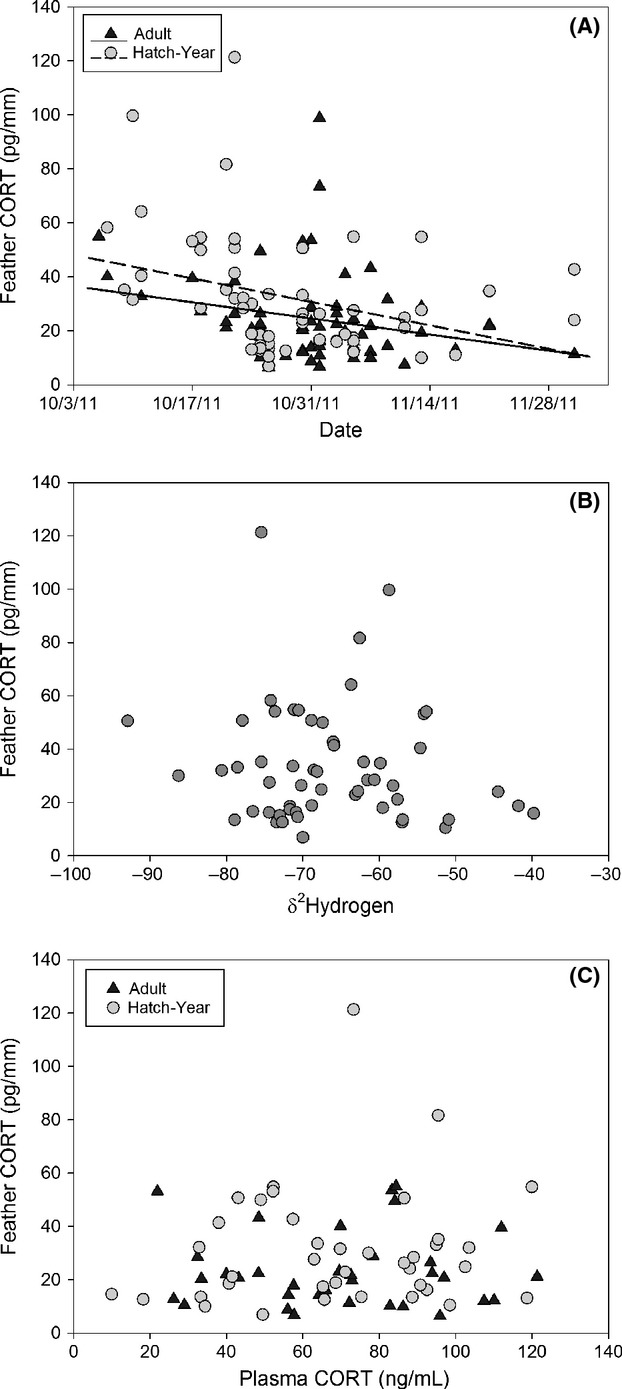
Relationship between corticosterone levels in the feathers of Northern saw-whet owls and the date of capture (A), *δ*^2^Hydrogen values (B), and plasma CORT levels (C). The triangle symbols are for adult owls, and the circles are for hatch-year owls; note that only the hatch-year owls are presented in plot B because of uncertainty in *δ*^2^Hydrogen values for adults.

**Figure 4:**
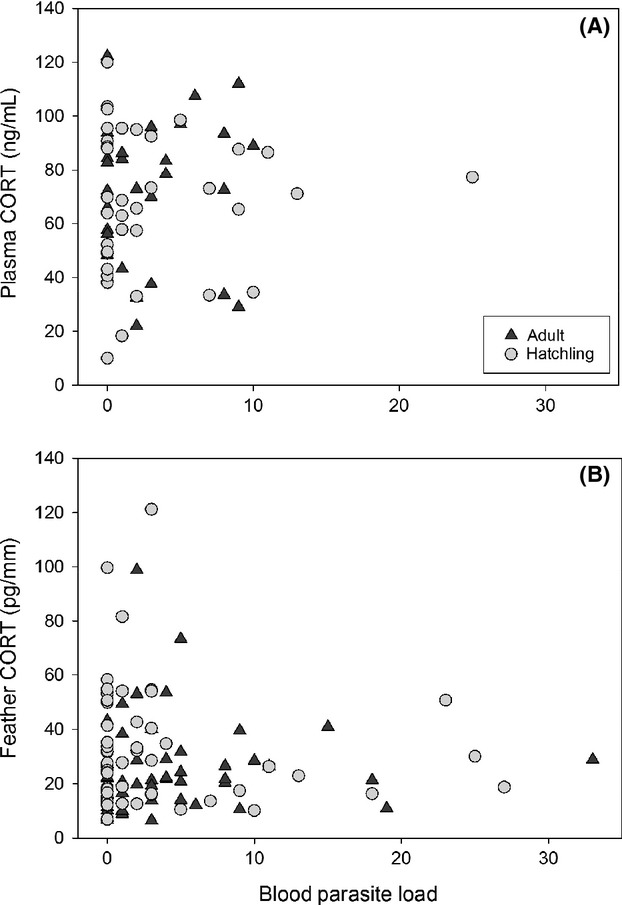
Plasma CORT levels of adult (triangles) and hatch-year (circles) Northern saw-whet owls in relationship to blood parasite loads. The triangle symbols are for adult owls, and the circles are for hatch-year owls.

Capture date was a strong predictor of CORT levels in feathers (Fig. 3A; *F*_1,123_ = 11.08, *P* = 0.001). Also, hatch-year owls tended to have higher CORT levels than adults (*F*_1,123_ = 3.67, *P* = 0.06); there was no interaction between age and date. There was no relationship between feather CORT and *δ*D values of hatch-year birds (Fig. 3B; *F*_1,53_ = 0.82, *P* = 0.37). Handling induced plasma CORT concentrations (*n* = 79) were unrelated to feather CORT levels (Fig. 3C; *F*_1,69_ = 0.69, *P* = 0.41) or capture date (*F*_1,75_ = 0.03, *P* = 0.87). It is important to note that the logistics of our netting operation prevented us from collecting baseline CORT values, our plasma data reflect handling induced concentrations (>3 min of researcher/trap exposure). Plasma CORT also did not vary between age classes; adults exhibited mean concentrations of 64.3 ± 0.1 ng/mL and hatch-year owls showed 63.0 ± 0.1 ng/mL. Five species of blood parasites were identified in these Northern saw-whet owls; the predominate parasite was a *Leucocytozoon* spp. (Table 1). Total parasite loads were not associated with plasma CORT (Fig. 4A; *F*_1,64_ = 1.63, *P* = 0.21), feather CORT levels (Fig. 4B; *F*_1,103_ = 0.74, *P* = 0.39), age (*F*_1,107_ = 0.01, *P* = 0.92), or date (*F*_1,107_ = 0.01, *P* = 0.92). In addition, body condition scores, based upon a wing chord/body mass index, were also not associated with CORT levels (feather CORT *F*_1,59_ = 0.02, *P* = 0.9; plasma CORT *F*_1,59_ = 0.07, *P* = 0.79) or total parasite loads (*F*_1,59_ = 0.1, *P* = 0.75).

**Table 1:** Blood parasite prevalence and mean intensity of infection (±SD) in Northern saw-whet owls

Parasites	Adult owls	Hatch-year owls
Haemoproteus	2% (1)	6% (4.8 ± 3.3)
Leucocytozoon	49% (6.2 ± 5.8)	34% (9.5 ± 12.9)
Microfilaria	8% (1.6 ± 0.9)	2% (2)
Plasmodium	8% (1)	9% (2.5 ± 3.2)
Trypanosoma	0	2% (1)
Mean parasite prevalence	58%	50%

## Discussion

The aim of this study was to illustrate how the integration of multiple biomarkers assayed on tissues collected from the same individual can shed light on whole-organism function across varying timescales. In feathers, we assayed deuterium (*δ*D) stable isotope values and glucocorticoids, while in blood we measured glucocorticoids and blood parasite levels. These coupled analyses span time periods ranging from weeks to seasons (Fig. 1). Through deuterium (*δ*D) stable isotope analysis, we found that fall-migrating Northern saw-whet owls caught in the Hudson Valley of New York use the Atlantic flyway and molted their prebasic feathers on breeding grounds in southern Canada (Fig2). These saw-whet owls overwinter in the southeastern United States (Beckett and Proudfoot 2011, 2012; De Ruyck et al. 2012). Through coupled glucocorticoid hormonal assays of these same feathers along with plasma, which integrate seasonal versus shorter-time intervals, we also found an association with higher corticosterone (CORT-primary avian glucocorticoid) levels in the feathers of owls that migrated earlier in the season. In contrast, we did not find associations between handling induced plasma CORT and feather CORT, or blood parasite loads. These patterns result from the hormonal kinetics and timing of deposition of CORT in varied tissues in response to environmental conditions (Bortolotti et al. 2008; Fairhurst et al. 2012, 2013b) and provide insight into how seasonal dynamics of ecological factors such as parasites affect the energetic workload of migratory birds (Deviche et al. 2001; Mougeot et al. 2010).

The pattern of elevated feather CORT in early migrating saw-whet owls likely reflects variance in migratory conditioning among the postbreeding and molting population, in which better conditioned or more rapidly prepared owls migrate early. Several studies suggest that elevation in plasma CORT is associated with increased foraging, body conditioning, migratory restlessness, and earlier timing of migration in birds ( Belthoff and Dufty 1998; Piersma; et al. 2000; Landys et al. 2004). Because the CORT found in feathers is an integrated result of plasma CORT levels during feather growth (Bortolotti et al. 2009; Fairhurst et al. 2013b), the elevated CORT that we found in the feathers of early migrating owls thus likely reflects increased energy allocated to preparation for rapid migration. A study by Lobato et al. (2010) also found an inverse relationship, similar to our results, between excreted CORT metabolites in uric acid and the arrival date of migrating flycatchers (Tyrannidae). Combined, these results suggest that CORT likely plays an important role in mediating preparation for migration as found by Landys-Ciannelli et al. (2002), and the timing of migration in owls and other birds as suggested by Lobato et al.'s (2010) results.

While feather CORT in the fall molt integrates the energetic workload of birds during the postreproductive and migratory preparation period (Landys-Ciannelli et al. 2002), recent work suggests that environmental conditions experienced during this period also influence CORT levels (Legagneux et al. 2013). Legagneux et al. (2013) found that feather CORT increased in association with late summer temperatures during prebasic molting in eiders (*Somateria* sp.) and suggested that this reflected a stress response to weather; directly through thermoregulation and indirectly through weather effects on food quantity and quality. In addition to weather, other environmental factors such as parasites can interact with an animal's life stage to effect CORT profiles. To explore these interactions, we also measured plasma CORT. Plasma CORT sampled during migration reflects short-term modulation of hormone levels and suppressed responses to environmental stressors to facilitate resource mobilization necessary to fuel costly migration, independent of short-term changes in energetic conditions (Schwabl et al. 1991; Holberton et al. 1996; Romero et al. 1997). We found no relationships between plasma and feather CORT, or parasite loads, which is perhaps a bit surprising, because parasites have been found to impose physiological stress and/or increase energetic demands (Deviche et al. 2001; Mougeot et al. 2010; Shurulinkov et al. 2012). However, like most trade-offs the effects of parasites on other life processes may only be apparent under compounding environmental stressors, like food shortages or poor health that create more variance in a population (Merino et al. 2000; Tomas et al. 2007; Mougeot et al. 2010). In addition, parasite loads vary seasonally and appear to impose a greater burden and fitness cost during spring migration and reproduction than during fall or other seasons (Deviche et al. 2001; Møller et al. 2003; Shurulinkov et al. 2012). Thus for these fall-migrating saw-whet owls, parasites did not appear to impose a detectable increase in CORT levels and thus may not represent a large energetic burden. Finally, the lack of a correlation between feather and handling induced plasma CORT highlights the fact that these tissues integrate differing time periods and reflect energetic demands during differing life stages.

### The future of integrated biomarkers

On the whole, these results illustrate the potential for integrating diverse biomarkers to assess interactions between environmental factors and animal health across varied time periods without the necessity of continually recapturing and tracking individuals. Biomarkers from research fields including stable isotope ecology, endocrine ecophysiology, nutritional ecology, and ecoimmunology are becoming more readily available and accessible, and when combined into an integrated framework hold great promise for advancing our understanding of environmental effects on animal health (Martin et al. 2011; Wagner et al. 2013; Milot et al. 2014). This approach could, for example, provide insight into the factors driving, as well as the impacts, of the boom and bust years in migratory densities of saw-whet owls (Whalen and Watts 2002; Brittain et al. 2009; Beckett and Proudfoot 2011). Applying this approach to a longitudinal study of migrating birds could also provide insight into how climate variability impacts the stress physiology and ecology of migrating animals. Indeed, several recent studies have demonstrated that such an integrated approach can provide insight into environmental effects on fitness-related traits including reproductive allocation (Kouwenberg et al. 2013), condition dependent responses to food availability (Fairhurst et al. 2013a) and long-term survival (Koren et al. 2012). In addition, other recent studies suggest that coupling diverse biomarkers may provide insight into microbiome and antimicrobial peptide interactions (Wellman-Labadie et al. 2007; Giraudeau et al. 2010) as well as host-parasite and disease interactions (Stapp and Salkeld 2009). While more work is necessary to standardize and make such diverse biomarkers more accessible to researchers, there is clearly great potential to broaden and deepen our understanding of environmental effects on animal performance, ecology, and health through integrating such biomarkers into comprehensive frameworks (Cohen et al. 2012; Wagner et al. 2013; Milot et al. 2014).
